# Principles and Methods for Improving the Thermoelectric Performance of SiC: A Potential High-Temperature Thermoelectric Material

**DOI:** 10.3390/ma17153636

**Published:** 2024-07-23

**Authors:** Yun Xing, Bo Ren, Bin Li, Junhong Chen, Shu Yin, Huan Lin, Jie Liu, Haiyang Chen

**Affiliations:** 1 School of Materials Science and Engineering, University of Science and Technology Beijing, Beijing 100083, Chinalinhgl@163.com (H.L.);; 2 Institute of Multidisciplinary Research for Advanced Materials, Tohoku University, 2-1-1 Katahira, Aoba-ku, Sendai 980-8577, Japan; 3 Advanced Institute for Materials Research (WPI-AIMR), Tohoku University, 2-1-1 Katahira, Aoba-ku, Sendai 980-8577, Japan

**Keywords:** SiC, thermal properties, electrical properties

## Abstract

Thermoelectric materials that can convert thermal energy to electrical energy are stable and long-lasting and do not emit greenhouse gases; these properties render them useful in novel power generation devices that can conserve and utilize lost heat. SiC exhibits good mechanical properties, excellent corrosion resistance, high-temperature stability, non-toxicity, and environmental friendliness. It can withstand elevated temperatures and thermal shock and is well suited for thermoelectric conversions in high-temperature and harsh environments, such as supersonic vehicles and rockets. This paper reviews the potential of SiC as a high-temperature thermoelectric and third-generation wide-bandgap semiconductor material. Recent research on SiC thermoelectric materials is reviewed, and the principles and methods for optimizing the thermoelectric properties of SiC are discussed. Thus, this paper may contribute to increasing the application potential of SiC for thermoelectric energy conversion at high temperatures.

## 1. Introduction

With the development of contemporary industry, energy and the environment have become important issues in sustainable human development. Waste heat, or radiant energy, makes up two-thirds of Earth’s energy emissions, which accelerate the pace of global warming [1,2,3]. Research on the reuse of waste heat is crucial for reducing the greenhouse effect and our dependence on fossil fuels due to carbon dioxide emissions. As functional materials with the ability to directly convert heat into electrical energy, thermoelectric materials have become a topic of interest for current research because they play a prominent role in energy reuse and environmental protection [4,5,6].

**Figure 1:**
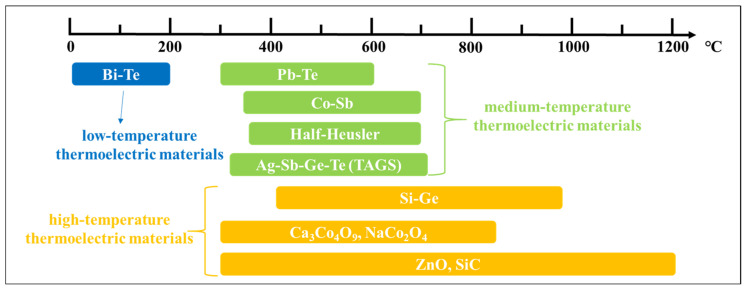
Typical operating temperature of various thermoelectric materials [14,15].

In nearly 200 years of research history, numerous thermoelectrics have been studied and classified according to their operating temperatures, as shown in Figure 1. These include low-temperature thermoelectric materials (25–300 °C), such as Bi_2_Te_3_; medium-temperature thermoelectric materials (300–700 °C), including PbTe, CoSb_3_, Ag-Sb-Ge-Te, and half-Heusler compounds; high-temperature thermoelectric materials (700–1000 °C), including Si_1-x_Ge_x_; oxide-based thermoelectric materials, such as NaCo_2_O_4_ and Ca_3_Co_4_O_9_; and certain wide-band semiconductor materials [7,8,9,10]. Low- and medium-temperature thermoelectric materials are commercially used in applications like electrical refrigeration and solar thermal power generation. However, the application of thermoelectric materials in fields requiring high temperatures has not been widely promoted because of the higher requirements for the use of temperature and high-temperature performance [11,12]. Researchers have conducted several studies on high-temperature thermoelectric materials, with NaCo_2_O_4_ and Ca_3_Co_4_O_9_ being two of the most studied materials [13]. They have the benefits of low cost and high thermal and chemical stabilities; however, their applicable temperatures (in the range of 300–900 °C) are not sufficiently high, as shown in yellow in Figure 1 [14,15]. ZnO has a wide range of applicable temperatures; however, its commercial application is impeded by intrinsic defects and p-type doping difficulties [16]. In addition, certain thermoelectric materials with narrow band gaps, such as Si_1-x_Ge_x_ [17], have intrinsic minority carriers in their valence bands (V_B_) that are prone to transitions at elevated temperatures, thus resulting in bipolar diffusion, i.e., both carriers contribute to heat transfer and become more pronounced with intrinsic excitations. These phenomena result in higher thermal conductivity, which can lead to thermoelectric property degradation. Therefore, developing a new type of thermoelectrical material that can operate stably at high temperatures is essential.

The emergence of wide-bandgap semiconductor materials provides a possible solution. These materials have a low possibility of bipolar diffusion and are particularly suitable for use at elevated temperatures. SiC, the most developed wide-bandgap semiconductor material, has the advantages of high mechanical strength, chemical and thermal stability, and corrosion resistance, and it is regarded as a promising material for high-temperature thermoelectrics. Compared with other oxide and alloy semiconductors (e.g., Bi_2_Te_3_, PbTe, and ZnO), SiC exhibits higher stability and better thermoelectric properties at temperatures exceeding 1000 °C [18]. Therefore, SiC is expected to be widely used for high-temperature thermoelectric power conversion.

The potential applications of thermoelectric devices utilizing SiC are vast and diverse. SiC-based thermoelectric devices can be employed to cool electronic components, significantly enhancing their performance and longevity [19]. They hold promise for use in refrigerators and air conditioners, offering an eco-friendly alternative to traditional cooling systems. Moreover, SiC-based thermoelectric devices can be utilized in both low and high-power applications. In low-power scenarios, such as wearable devices and sensors, their ability to harness waste heat and convert it into electrical energy provides a unique energy source. In high-power applications, SiC’s high-temperature stability and efficient energy conversion make it an attractive choice for industrial waste heat recovery and automotive exhaust systems [20,21,22].

Furthermore, SiC-based thermoelectric devices can serve as thermal energy sensors, accurately measuring temperature gradients and enabling precise temperature control in various industries. Aerospace applications, particularly in supersonic vehicles and rockets, stand to benefit greatly from SiC’s combination of high-temperature stability, mechanical strength, and thermoelectric performance, offering innovative solutions for power generation, cooling, and thermal management.

This work presents a systematic review of the state of the field. Performance optimization methods and future development trends of SiC thermoelectric materials are discussed to provide a reference for promoting its application in the field of high-temperature thermoelectric energy conversion.

## 2. Introduction to SiC

According to the classification of crystal type, as many as 250 polytypes of SiC crystals exist. SiC can be divided into α (hexagonal) and β (cubic) crystal types. α-SiC can be divided into more than 100 types of polytypes, such as 2H, 4H, and 6H. Only β-SiC, also known as 3C-SiC, belongs to the cubic crystal system of polytypes. Additionally, β-SiC is the low-temperature-stabilized phase, and α-SiC is the high-temperature stabilized phase. Particularly, the β-SiC phase is more stable at temperatures less than 2000 °C and gradually undergoes a transformation to the α-SiC phase when the temperature exceeds 2000 °C [23]. During the sintering process, the addition of liquid-phase sintering additives promotes the β-to-α phase transition, and the phase transition temperature is lower than 2000 °C [24,25].

**Figure 2:**
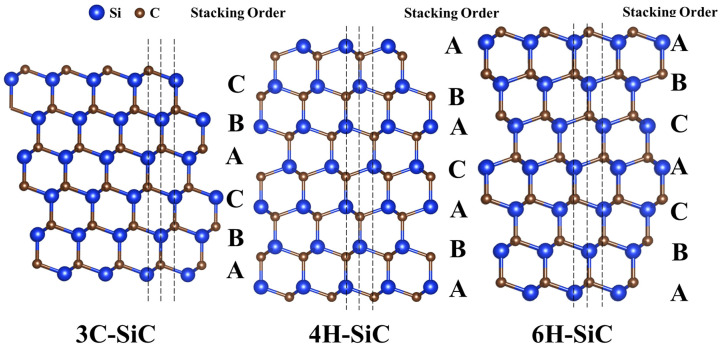
Stacking order of different SiC polytypes [26].

Common SiC crystal phases include 4H, 6H, and 3C. The crystal structure of SiC comprises three atomic layers: A, B, and C, corresponding to various close-packed positions. The stacking order of 4H-, 6H-, and 3C-SiC is shown in Figure 2 [26]. Although different polytypes differ only in the stacking order of the atomic layers, they exhibit different electronic structures and performance. A comparison of the structures and properties of 4H-, 6H-, and 3C-SiC is presented in Table 1 [27,28]. In addition to a small dielectric constant, high carrier saturation drift rate, high critical breakdown electric field strength, excellent radiation resistance, and good thermal stability, SiC has a large forbidden bandwidth, which is its most distinctive feature [29]. The space group, cell parameter a, and bandgap of cubic 3C-SiC are F-43m, 0.4349 nm, and 2.3 eV, respectively. Conversely, the hexagonal 4H- and 6H-SiC have a cell parameter a of 0.3073 nm, cell parameter c of 1.0053 and 1.5118 nm, and bandgap of 3.2 and 3.0 eV, respectively. Table 1 shows that the electrical properties of different SiC crystal phases significantly differ due to variations in the stacking order and bandgap. For example, 3C-SiC has a significantly smaller energy gap width than 4H- and 6H-SiC, indicating a higher intrinsic carrier concentration and a saturated electron migration rate, thereby resulting in better electrical conductivity. The resistivity of SiC ceramics prepared using 3C-SiC powders (3.0 × 10^−10^–8.32 × 10^−2^ Ω·m) is lower than that of SiC ceramics prepared using α-SiC powders (5.4 × 10^−2^–1.2 × 10^16^ Ω·m) [30]. Thus, 3C-SiC is more suitable than α-SiC for application as a thermoelectric material.

From a technical economic perspective, SiC possesses several advantages that make it an attractive choice for thermoelectric applications. Firstly, SiC’s abundant raw material elements ensure a stable supply chain and reduce material costs compared to some rare-earth-based thermoelectric materials. Secondly, its high thermal and chemical stability, combined with good mechanical strength, allows for a longer service life and reduced maintenance costs in harsh environments. Furthermore, the use of SiC-based thermoelectric materials can contribute to energy savings and emission reductions by improving energy conversion efficiency, aligning with the global trend toward sustainable energy solutions. The economic potential of SiC in the thermoelectric field is further enhanced by ongoing technological advancements that aim to reduce production costs and improve scalability.

## 3. Methods for Improving Thermoelectric Properties

The thermoelectric figure of merit (*ZT*) is a key criterion for describing the thermoelectric properties of a material, and it is defined and calculated using the following formula:

(1) $$ZT=S^{2}\sigma T/\kappa$$

where *S* is the Seebeck coefficient; *σ* is electrical conductivity; *κ* is thermal conductivity; and *T* is Kelvin temperature. According to the equation, a high-performance thermoelectric material must have high *S* and *σ* values and a low *κ* value.

**Figure 3:**
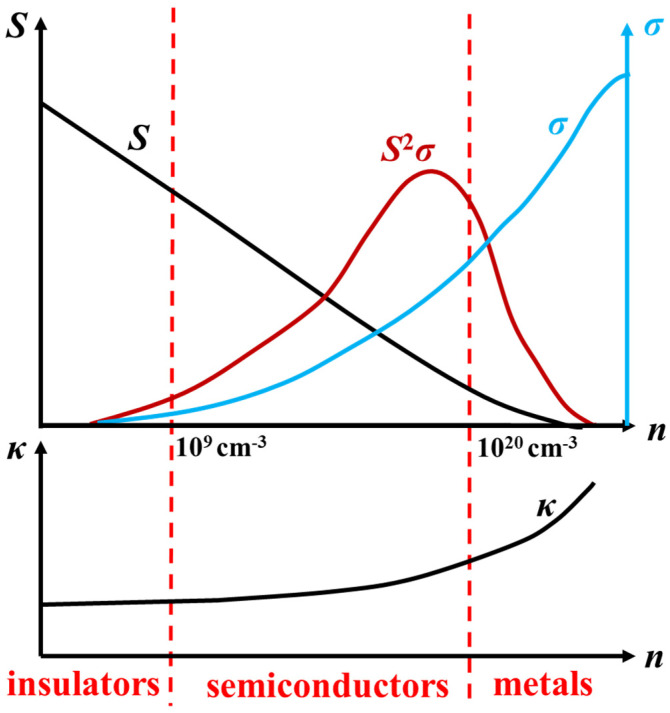
Dependence of *S*, *σ*, and *κ* on carrier concentration [14,15].

Figure 3 shows the influence of carrier concentration on various thermoelectric performance parameters. In semiconductor thermoelectric materials, *S*, *σ*, and *κ* act in conjunction and are all related to the carrier concentration. When the concentration of carriers is minimal at 10^8^ cm^−3^, the Seebeck coefficient is high; however, the electrical conductivity is low and thermoelectric properties are poor. Elevating the concentration of charge carriers results in improved electrical conductivity and a reduction in the entropy differential between surfaces at high and low temperatures, resulting in a lower Seebeck coefficient. Based on existing research, the optimum value of carrier concentration is typically 10^19^–10^20^ cm^−3^ in the heavily doped state for most semiconductor materials. Since the scattering mechanisms of electrons and thermally conductive phonons interact with each other, a decrease in thermal conductivity often affects conductivity and the Seebeck coefficient [31,32,33]. Thus, the three important parameters (*S*, *σ*, and *κ*) for the thermoelectric properties of materials are closely interrelated. Moreover, attempts to increase or decrease one of these parameters often result in non-synergistic changes in the others, hindering improvements in thermoelectric properties. Thus, one of the long-standing objectives in thermoelectric material science is to achieve the independent or synergistic regulation of electrical and heat transport.

**Figure 4:**
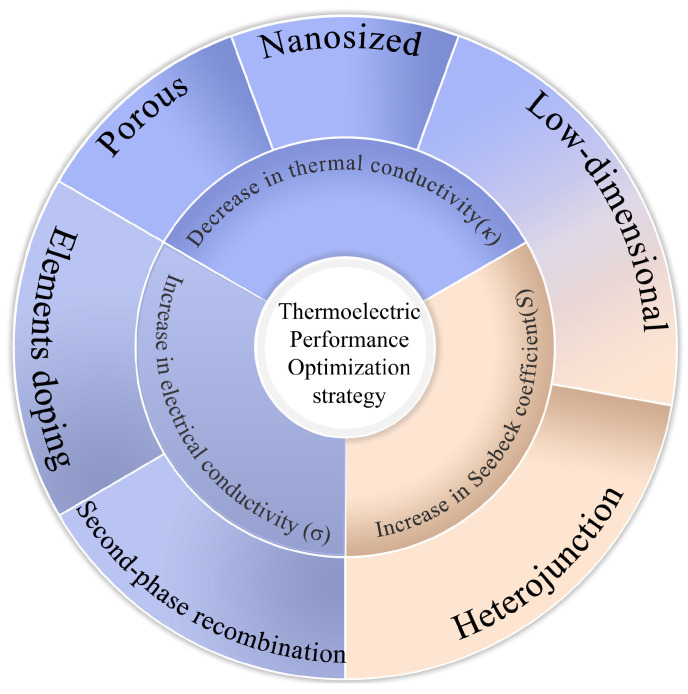
Strategies for optimizing the thermoelectric properties of SiC.

Several investigations have been conducted on improving thermoelectric properties. Enhancing electrical conductivity primarily depends on elemental doping and second-phase recombination to achieve an increase in the carrier concentration. Reducing thermal conductivity is primarily based on the reduction in lattice thermal conductivity via low-dimensionalization, nanosizing, and increased porosity [34,35,36]. The Seebeck coefficient can be increased by regulating the energy-band structure to control the carrier concentration and effective mass of the density of states (DOS) [37]. In the process of optimizing the performance of thermoelectric materials, doping is often used to bring the thermoelectric material to an optimum carrier concentration and achieve an increase in electrical conductivity. The lattice thermal conductivity is reduced by adjusting the phonon dispersion relationship via phonon engineering, and the Seebeck coefficient is increased by changing the DOS effective mass through energy-band engineering (Figure 4). The synergistic optimization methods of *S*, *σ*, and *κ* will be further discussed from the perspective of SiC thermoelectric parameter optimization.

SiC, with its unique band structure and adjustable energy gap through doping, exhibits an enhanced Seebeck coefficient and electrical conductivity, which are vital for optimizing the power factor. Additionally, the charge carrier concentration and mobility play pivotal roles in determining the electrical transport properties. In SiC, careful control of these parameters through doping strategies and nanostructuring has been shown to improve thermoelectric performance. Other properties, although not directly contributing to the thermoelectric figure of merit, are equally important for material selection and device design. The diffusion properties of SiC ensure stable performance over extended periods, while its low oxidizability and high mechanical strength (including compression and shear strength) make it suitable for harsh environments. Furthermore, the coefficient of thermal expansion (CTE) of SiC is well suited for integration with other materials, reducing thermal stress and improving device reliability.

### 3.1. Increase in Electrical Conductivity

The electrical conductivity (*σ*) is an inherent feature of a material that expresses its capacity to conduct electricity. For instance, some materials, like alloys, have a comparatively high electrical conductivity in their unprocessed state, while others, like SiC, have a relatively low electrical conductivity. The formula below is used to calculate electrical conductivity:

(2) σ=nqμ

where *n* is the carrier concentration, *q* is the carrier charge, and *μ* is the carrier mobility. Thus, a high *σ* requires a high carrier concentration and high mobility. The *ZT* can be improved via doping and with a two-phase composition to increase the conductivity [38].

#### 3.1.1. Element Doping

Element doping is generally substitutive; thus, the doping elements replace the atoms in the lattice. The solid’s solubility restricts how much a SiC lattice may be doped. When this limit is exceeded, the excess dopant material cannot enter the lattice and exists as a second phase at the grain boundaries. This phenomenon, known as dopant saturation, is crucial in understanding the limits of doping effectiveness and the resultant material properties. The electronic configurations and chemical bond structures of dopants determine whether they function as acceptors or donors in the SiC lattice. Specifically, acceptor dopants, such as B, Al, and Sc, introduce holes (positive charge carriers) into the valence band, while donor dopants like N contribute electrons (negative charge carriers) to the conduction band. The effects of N, V, Al, B, and Sc on the resistivity of porous SiC ceramics were studied by Kultayeva et al. [39].

With a minimal difference in porosity, the study revealed a clear distinction in resistivity based on dopant type. The lowest resistivity was found for SiC ceramics doped with the n-type dopant N (2.1 × 10^−3^ Ω·m), indicating a high electron mobility contributed by the N dopant. This was followed by the p-type dopants B, Sc, and Al (0.69, 1.5, and 3.9 Ω·m, respectively), the higher resistivities of which reflect the presence of holes as charge carriers, which generally have lower mobility than electrons. The highest resistivity was observed for V-doped SiC ceramics (4.2 Ω·m), possibly due to V’s unique electronic configuration that does not significantly contribute to either electron or hole generation or to its tendency to form defects that hinder charge transport.

N-type doping results in higher electron mobility and lower resistivity than p-type doping, as electrons are more mobile than holes in many semiconductor materials [39]. The p-type dopants commonly used for SiC are Al, B, Ag, Ga, Ni, Cu, and Sc, whereas the commonly used n-type dopants are N, P, Co, and Fe. A reasonable selection of doping elements and concentrations can increase carrier concentration, improve the conductivity of SiC materials, and yield p- or n-type semiconductors with good performance.

##### N-Type Doping

(1) N doping

**Figure 5:**
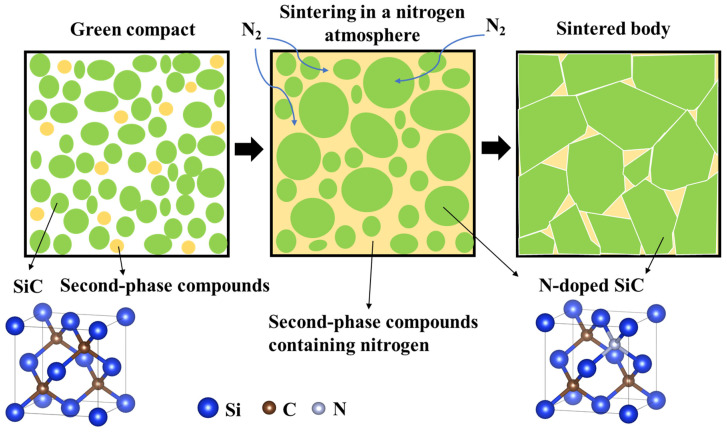
Diagrammatic representation of the N-doping process in SiC ceramics in a N_2_ environment [40].

Nitrogen is widely used as an n-type dopant for SiC. In the N doping of SiC, the N atom preferentially occupies the position of the C atom. Because five valence electrons are present in the outermost layer of the N atom, four create covalent bonds with the four Si atoms in their immediate vicinity, freeing up one valence electron. When this electron is ionized, it transitions from the bound state to the conduction band (C_B_) and becomes a conducting electron, thereby significantly increasing the conductivity. A doped element that releases electrons and produces conducting electrons is known as an n-type dopant. To achieve N doping, two methods are commonly employed: sintering in a N_2_ atmosphere and adding N-containing compounds. Both specific doping processes are shown in Figure 5 [40]. First, the SiC powders and second-phase compounds (sintering additives or N-containing compounds) are evenly mixed and then sintered in a N_2_ environment. During the sintering process, the N in the N_2_ gas dissolves in the liquid-phase sintering additives, providing part of the N source, and the N-containing compounds also release N. Finally, with the growth of SiC grains following dissolution–recrystallization, N atoms dissolve in the SiC lattice, and N doping is achieved. Kitagawa, Noda, and Kado et al. [41,42,43] prepared β-SiC doped with 3–10 wt% Si_3_N_4_ using spark plasma sintering at 2000 °C and 50 MPa. The relative density of the sintered material exceeded 85%. Both the Hall and Seebeck coefficients were negative, indicating that N functioned as a donor in SiC and that most carriers were electrons. With an increasing Si_3_N_4_ content, the carrier concentration increased, which resulted in a decrease in the Seebeck coefficient and an increase in conductivity. When the doping amount was 7 wt% at 700 °C, the maximum power factor and carrier concentration were 1.5 × 10^−4^ W/mK^2^ and 1.7 × 10^26^ m^−3^, respectively.

(2) Fe doping

INAI [44] successfully prepared n-doped SiC with a negative Seebeck coefficient by sintering β-SiC and 1–20 wt% Fe under an Ar atmosphere. In their study, the Fe atoms were completely integrated into the SiC lattice and did not form a second phase. The addition of Fe significantly inhibited the conversion of 3C-SiC to 6H-SiC. With increasing Fe content, the grain size of SiC gradually decreased, and the particle shape changed from square to round, forming a porous ceramic structure. Because Fe atoms were doped into the SiC lattice and provided numerous carriers, the reduction in resistivity was significant. For the sample doped with 10 wt% Fe, the resistivity decreased to 1.8 × 10^−4^ Ω·m. At approximately 750 °C, the *ZT* of the sample doped with 20 wt% Fe was at its maximum (approximately 1.023 × 10^−2^). Zhang [45] provided an in-depth explanation for the mechanism of n-type semiconductor formation achieved via doping SiC with Fe atoms. Because the atomic radius of the Fe atom (1.26 Å) differs from that of the C atom (0.77 Å) and is less than that of the Si atom (1.18 Å), Fe atoms could easily replace Si atoms. The outer electron layer of an Fe atom is arranged as 3d^6^4s^2^, indicating that it contains six 3d and two 4s electrons. The calculated results showed that when Fe replaced Si, a p–d hybrid effect occurred between the 3d electrons of the Fe atom and the p-orbital electrons of the C and Si atoms [46,47]. This hybrid effect caused the Fermi energy levels to shift toward the C_B_, providing donor energy levels within the forbidden band, thus forming n-type semiconductors.

##### P-Type Doping

(1) Al doping

Noda and Kado et al. [42,43] prepared β-SiC doped with 3–10 wt% Al_4_C_3_ via spark plasma sintering at 2000 °C. The prepared samples had a density of up to 95%. X-ray diffraction (XRD) results showed that Al was successfully doped into the SiC lattice and that it promoted the phase transition from β-SiC to α-SiC. When the doping amount was 7 wt% at 700 °C, the power factor attained a maximum of 2 × 10^−5^ W/mK^2^, and the carrier concentration was 7.6 × 10^24^ m^−3^. However, the sample performed slightly worse than the sample doped with Si_3_N_4_ under the same conditions. This may be explained by the deep acceptor energy level of Al in SiC, which resulted in certain changes that affected its properties [48]. In addition, with increased temperature, the conductivity and Seebeck coefficient of the samples increased. Therefore, their material exhibited good thermoelectric conversion performance under high-temperature conditions.

(2) Ag doping

By supplying a carrier concentration 10^3^–10^4^ times larger than that of SiC with typical doping concentrations of Al, Ag is a highly effective dopant for decreasing the resistivity of p-type SiC [49,50]. Shrader et al. [51] explained the mechanism of Ag-doped SiC via calculations. Ag tends to occupy the Si position rather than the C atom. This is because a Ag atom has an atomic radius (1.44 Å) comparable to that of a Si atom (1.18 Å) and greater than that of a C atom (0.77 Å) [51]. In addition, the Ag atom has only one valence electron; thus, the single valence electron forms a covalent bond with one electron of a C atom in the Si_1-x_Ag_x_C structure, creating holes. In order to gain a better understanding of the impact of Ag doping on the thermoelectric properties of SiC, Kato et al. [52] carried out a number of investigations. Their study revealed that Ag doping did not significantly affect the XRD patterns of the samples, indicating that the Ag atoms were successfully incorporated into the lattice structure of SiC without causing significant structural changes. During preparation, the sample was sintered at 2100 °C for 2 h under normal pressure. Owing to the volatilization of the additives during the sintering process, the sample formed a porous structure with hole diameters ranging from 0.1 to 10 μm. When the Ag doping concentration was 2 wt%, the *ZT* value of the sample at 700 °C reached a maximum value of 0.3892 and the carrier concentration was 2.3 × 10^26^ m^−3^. This result confirmed that Ag is a highly effective dopant for reducing the resistivity of SiC thermoelectric semiconductors.

##### Element Co-Doping

Improving the thermoelectric properties of materials remains limited when using a single doping element [41,42,43,44,52]. Thus, to further optimize thermoelectric properties, several studies have focused on the synergistic effects of multicomponent doping. Chul-Hoon PAI [53] compared the thermoelectric characteristics of SiC doped with AlN and Al_4_C_3_. Following sintering at 2150 °C for 3 h, porous SiC ceramics with a relative density of 50–60% were obtained. XRD analysis showed that these samples contained no impurities other than SiC, confirming that Al and N were fully integrated into the SiC lattice. The AlN-doped samples exhibited significantly higher Seebeck coefficients owing to the compensation of acceptor (Al) and donor (N). However, the conductivity of the AlN-doped sample was lower than that of the Al_4_C_3_-doped sample owing to the annihilation effect of the carriers. With increasing AlN content, SiC underwent a reverse phase transition from 6H to 4H, which further resulted in an increase in carrier concentration. In the abovementioned study, the effect of the Seebeck coefficient was greater than that of the conductivity; therefore, the *ZT* of AlN-doped SiC was higher than that of the Al_4_C_3_-doped SiC. When the AlN doping amount was 3 wt%, *ZT* attained a maximum of 1.25 × 10^−2^.

Ryol et al. [54] calculated the activation energy, which increased when the AlN doping in SiC was greater than 50 mol%. This resulted in a larger forbidden bandwidth and sharp decrease in conductivity. The activation energy was calculated as follows:

(3) $$\log\sigma =-\frac{0.434E_{g}}{2K_{B}T}+\log\sigma_{0}$$

where *σ* stands for electrical conductivity, *T* for thermodynamic temperature, *K_B_* for the Boltzmann constant, and *E_g_* for activation energy.

#### 3.1.2. Second-Phase Recombination

Element-doping methods have shown good results in previous studies; however, the available types of doping elements are relatively limited [41,42,43,44,52]. Adding second-phase materials is an effective strategy for optimizing the electrical conductivity of SiC [55,56]. SiC ceramics doped with 1–40 wt% Ge were prepared and analyzed using XRD [56]. The results showed that the added Ge existed in the sample as a secondary phase. With an increase in Ge content, the bulk density of the samples decreased to 55%, which was lower than that of traditional sintered SiC ceramics. At approximately 750 °C, the resistivity of the sample with 40 wt% Ge was 5 × 10^−4^ Ω·m, which was 1/20 of that of the sample with 1 wt% Ge. In addition, the thermal conductivity of the lattice was reduced by mass fluctuations because Ge is a heavy atom. Thus, the properties of SiC ceramics can be optimized by adding a second-phase material.

The thermoelectric properties of SiC ceramics doped with various dopants are summarized in Table 2, where the *PF* of the N-doped samples is far greater than that of the Al-doped samples under the same sintering conditions and at the same doping concentrations. N-type doped electrons have a higher mobility and lower resistivity than p-type doped holes [39]. In addition, the synergistic effect of multi-element doping is more prominent than that of single-element doping for controlling the thermoelectric parameters. In addition to the type and proportion of the doping elements, the sintering method has a significant effect on the thermoelectric properties of SiC ceramics. Sintering directly affects the microstructures of SiC ceramics, such as the grain-boundary structure, grain size, and porosity. These changes in microstructural characteristics further affect basic parameters such as carrier concentration and thermal conductivity, thus substantially affecting the thermoelectric properties of SiC ceramics. Therefore, the choice of doping element and sintering method determines the thermoelectric properties of SiC.

### 3.2. Decrease in Thermal Conductivity

Heat conduction in semiconductors is primarily achieved via carrier motion and lattice vibrations. The expression for thermal conductivity in a semiconductor is

(4) $$\kappa =\kappa_{e}+\kappa_{l}$$

where *κ_e_* is the contribution of carrier motion to the thermal conductivity and *κ_l_* is the thermal energy transfer due to vibrations and mutual coupling of the lattice atoms.

The carrier thermal conductivity, *κ_e_*, follows the Wiedemann–Franz law [57]:

(5) $$\kappa_{e}=L\sigma T$$

where *L* is the Lorentz constant, *σ* is the electrical conductivity, and *T* is the thermodynamic temperature. The lattice thermal conductivity is calculated as follows:

(6) $$\kappa_{l}=-\frac{1}{3}C_{v}vl$$

where *C_v_* is the specific heat at a constant volume, *v* is the velocity of motion of the phonon, and *l* is the average free distance of the phonon between scatterings [58]. The magnitude of the average free range of the phonons is determined by the scattering mechanism of the phonons in the crystal. In general, the contribution of lattice vibrations (phonons) to the thermal conductivity is larger than that of carriers [58]. Moreover, the lattice thermal conductivity is an independent parameter; therefore, to maximize the characteristics of thermoelectric materials, one key strategy is to modulate the lattice thermal conductivity. An accurate description of the lattice thermal conductivity requires solving the complex Boltzmann equation for phonons. Therefore, the Debye model is typically used to represent the lattice thermal conductivity of a material, as follows:

(7) $$\kappa_{l}=\frac{K_{B}}{2\pi^{2}v}{\left(\frac{K_{B}T}{\hbar }\right)}^{3}\int_{0}^{\theta_{D}/T}\frac{x^{4}e^{x}}{{\tau_{c}^{-1}(e^{x}-1)}^{2}}dx$$

where *x = ℏɷ/K_B_T*, *ɷ* is the phonon frequency, *θ_D_* is the Debye frequency, *τ_c_* is the relaxation time, *K_B_* is the Boltzmann constant, and *ħ* is the Planck constant. The relaxation time *τ_c_* in the above equation is the result of the combined effect of multiple scattering mechanisms and can be expressed as [59,60]

(8) $$\frac{1}{\tau_{c}}=\frac{1}{\tau_{B}}+\frac{1}{\tau_{U}}+\frac{1}{\tau_{D}}+\ldots$$

where *τ_B_* corresponds to grain-boundary scattering (B), *τ_U_* to phonon–phonon scattering (U), and *τ_D_* to scattering from crystal defects (D). Certain scattering mechanisms have negligible effects on the thermal conductivity, which is not discussed in depth in this paper.

(1) Grain-boundary scattering

Due to boundary scattering’s independence from frequency and the significance of its contribution at low temperatures, the relaxation time for grain-boundary scattering is only related to the average rate of the phonon *v_s_* and average size of the grains *L*, expressed as

(9) $$\frac{1}{\tau_{B}}=\frac{v_{s}}{L}$$

(2) Phonon–phonon scattering

There are two modes of phonon–phonon scattering. In the first N-process (at low temperatures), new phonons are produced following phonon collisions that do not extend beyond the Brillouin zone. Moreover, the energy flow direction remains unchanged, and no thermal resistance is generated. In the second U-process (inversion of the phonon obtained following the high-energy phonon collision beyond the Brillouin zone), the relaxation time of the scattering can be approximated as follows:

(10) $$\tau_{U}^{-1}=\frac{\gamma^{2}}{Mv_{s}^{2}\theta_{D}}\omega^{2}T\exp\left(-\frac{\theta_{D}}{T}\right)$$

where *M* is the average atomic mass of the crystal and *γ* is the Greene Eisen constant. The probability of the process proceeding depends only on the number of phonons in the crystal, which is proportional to the temperature.

(3) Point-defect scattering

The effect of point-defect scattering on the lattice thermal conductivity of materials is significant, and it can reduce the lattice thermal conductivity.

(11) $$\tau_{D}^{-1}=\frac{V}{4\pi v_{s}^{3}}\omega^{4}\sum f_{i}{\left(\frac{\bar{m}-m_{i}}{\bar{m}}\right)}^{2}$$

Here, *V* is the average volume of atoms, *f_i_* is the proportional fraction of atoms with mass *m_i_*, and $\bar{m}$ is the average mass of atoms.

At low temperatures, low-frequency, long-wave phonon scattering is extremely weak when the scattering of phonons via grain boundaries dominates. Therefore, increasing the number of dislocations within the crystal or reducing the grain size can reduce thermal conductivity. As the temperature increases, the phonons in the lattice are transformed into high-frequency, short-wave phonons because they bear the main heat. The introduction of point defects can effectively lower the lattice thermal conductivity, and these phonon–phonon and lattice defects are important in scattering phonons. The experiment by Ioffe [61] and the theory of Keyes [62] demonstrate that lattice thermal conductivity decreases as the average atomic weight increases. Therefore, heavy-element doping can reduce the thermal conductivity of a material and improve the thermoelectric properties of SiC.

#### 3.2.1. Porosification

Lowering the heat conductivity is a crucial problem in SiC thermoelectric material research. One way to reduce *κ_l_* is to dope it with heavy elements to increase the scattering of short-wave phonons. However, when the lattice thermal conductivity is reduced to a certain level, the contribution of long-wave phonons to the thermal conductivity cannot be ignored. Long-wave, low-frequency phonons carry large quantities of heat, are more likely to be dispersed near the grain boundaries, and have a lengthy free range [63]. Therefore, another way to reduce *κ_l_* is to introduce more grain boundaries or interfaces into the material. Because grain boundaries or interfaces have a scattering effect on phonons, phonon thermal conductivity can be reduced. The interface reduces thermal conductivity, while affecting the electrical conductivity to a lesser extent; thus, the thermal conductivity can be independently regulated [64].

**Figure 6:**
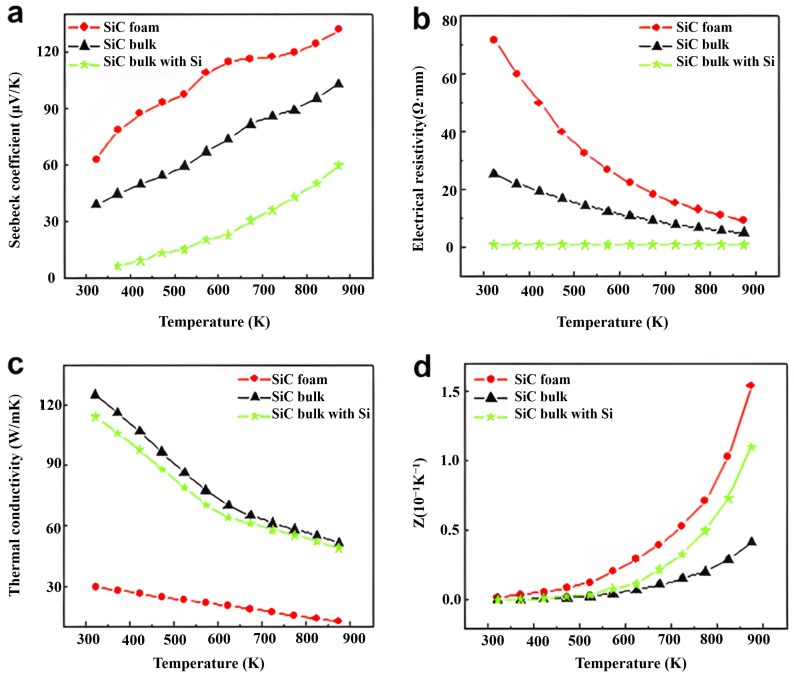
Thermoelectric characteristics of variously structured SiC ceramics in relation to temperature: (**a**) *S*, (**b**) *ρ*, (**c**) *κ*, and (**d**) *Z* [69].

An effective method for increasing the count of interfaces in a material is to increase the porosity of the thermoelectric material [65,66]. Thus, high-porosity nanomaterials have higher *ZT* values [67,68]. Wei et al. [69] investigated the thermoelectric properties of SiC foam and bulk ceramics doped with 5 wt% Si. The results of the experiment are presented in Figure 6. The Seebeck coefficient of the SiC foam ceramics was higher than that of the SiC bulk ceramics. Moreover, the resistivity of the SiC foam was higher at low temperatures. Conversely, the difference in resistivity between the two samples was negligible at high temperatures owing to the dominant role of intrinsically conducting increased carrier concentrations. The 3D mesh macrostructures were effective in reducing the thermal conductivity. For instance, the bulk ceramics’ thermal conductivity was four times greater than that of the foam ceramics. Moreover, at 873 K, the *ZT* value of the SiC foam was 1.338 × 10^−4^, which was higher than that of the bulk ceramic (3.65 × 10^−5^). Despite the high resistivity of the SiC foam, its Seebeck coefficient significantly increased while its thermal conductivity significantly decreased; thus, compared to bulk ceramics, the porous SiC foam exhibited superior thermoelectric capabilities.

Currently, porous SiC thermoelectric materials are primarily fabricated from biological materials, and researchers have used a bionic approach with loose porous wood as a template. This method not only preserves the complex structure of the C template obtained via the pyrolysis of wood but also enables the material to have better mechanical, thermal, and electrical properties than ordinary ceramics. The specific pore structure of a biological template results in conductivity anisotropy.

The electrical conductivities of porous SiC/Si ceramic composites prepared from bamboo were studied by Mallick et al. [70]. The conductivities measured parallel to (*σ*_║_) and vertical to (*σ*_⊥_) the natural plant growth axis were anisotropic (anisotropy~2), while the Seebeck coefficient was almost isotropic. B’ejar et al. [71] obtained SiC/Si composites via the pyrolysis of prefabricated bodies of pine and beech wood infiltrated with molten Si and explored the conductive properties of the materials. Maeda et al. [72] successfully prepared β-SiC/Si from rice hulls by impregnating them with an FeSO_4_ solution and carbonizing them. They then examined the impact of nitrogen on the thermoelectric properties of the material using hot-press sintering at 2100 °C with nitrogen. The investigation determined the *PF* to be 4 × 10^−6^ W/mK^2^, thermal conductivity to be 8 W/mK, and *Z* to be 5.5 × 10^−5^ K^−1^ for the material. Using a pulsed current sintering apparatus, Fujisawa et al. [73] created SiC/C composites from a combination of silica powder and charcoal. A maximum *ZT* of 1.6 × 10^−4^ was obtained at 200 °C.

#### 3.2.2. Low Dimensionality

The low-dimensionalization of existing bulk thermoelectric materials can substantially improve their thermoelectric properties. Theoretical simulations by Hicks et al. [74] on the thermal conductivity of Bi_2_Te_3_ 2D-layered materials revealed that the thermal conductivity of the material dramatically dropped as thickness decreased. In addition, if a nanoscale superlattice with multiple layers of different orientations can be prepared, a *ZT* value 10 times higher than that of the bulk material can be obtained, reaching 6.9 at room temperature. The applications of such high-performance thermoelectric materials have the potential to revolutionize. Theoretical analysis showed that 1D quantum lines and 0D quantum dot structures have better thermoelectric properties than superlattices. Thus, low-dimensional thermoelectric materials are important directions for the growth of high-performance thermoelectric materials. Theoretical and experimental studies have shown that the thermoelectric properties of low-dimensional systems are superior to those of the corresponding bulk materials. This is partly because the increased surface area of low-dimensional materials enhances the interfacial scattering of phonons, thus reducing lattice thermal conductivity. In low-dimensional systems, the quantum confinement effect elevates the DOS close to the Fermi energy level, which raises the Seebeck coefficient.

##### SiC Films

In traditional thermoelectric parameter measurements of bulk materials, the thermocouple and voltage measurement probes are millimeters in size. When these devices measure low-dimensional nanostructured materials, errors in accuracy due to size mismatches present a significant issue. Lei et al. [75] developed a high-precision micro-electro-mechanical systems device to solve this problem. The device could measure the conductivity, thermal conductivity, and Seebeck coefficient of low-dimensional samples all at once, thereby avoiding errors caused by changing the samples. Because the test device was suspended, several heat transfer channels were isolated during the test, which improved the measurement accuracy of the thermal conductivity of the samples. The device was used to measure the thermoelectric parameters of heavily doped n-type poly-SiC thin films. The transverse thermal conductivity of the polycrystalline SiC films (64 W/mK) was significantly lower than that of bulk SiC (270 W/mK) owing to the increase in interface and impurity scattering (Table 3) [75,76,77]. This discovery was important to understand the thermoelectric properties of low-dimensional nanostructured materials.

##### SiC Fibers

Papanikolaou et al. [78] simulated lattice heat transport in SiC nanowires and bulk materials with cross-sectional areas of 4–17 nm^2^. Their calculation demonstrated that SiC nanowires have a lower thermal conductivity than bulk SiC. They also analyzed the effect of cross-sectional and surface defects on heat transport and found that different surfaces resulted in a 20% change in the value of *κ*.

**Figure 7:**
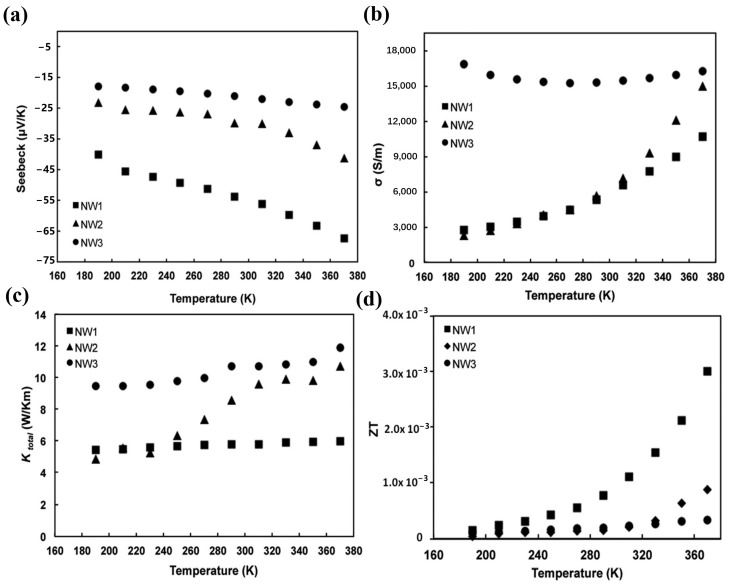
Thermoelectric parameters of each nanowire as a function of temperature: (**a**) *S*, (**b**) *σ*, (**c**) *κ*, and (**d**) *ZT* [79].

Valentín et al. [79] measured the thermoelectric parameters of three SiC nanowires in the temperature range of 190–370 K using a suspended micro-resistant temperature measuring device (Figure 7). The three SiC nanowire samples were named NW1, NW2, and NW3. As shown in Figure 7, significant differences in the thermoelectric properties of the three nanowires were observed. These differences may be due to the different crystal structures of each nanowire and the different scattering of defects, such as boundaries, impurities, and dislocations, in different nanowires. The thermal and electrical conductivities of the nanowires were affected to some extent. For all samples, the temperature dependency of the Seebeck coefficient is displayed in Figure 7a, where the Seebeck coefficient ranges from 18 to 68 μV/K. The outcomes corresponded with the information obtained from several β-SiC polycrystalline films [80]. The thermal conductivities, which ranged from 4 to 12 W/mK and were significantly lower than the reported values of bulk materials at ambient temperature (~300 W/mK) [81], are displayed in Figure 7c. This indicates the impact of defect scattering and phonon surface scattering. The *ZT* values for each sample are displayed as a function of temperature in Figure 7d. *ZT* increased with temperature, and NW1 was 3.01 × 10^−3^, NW2 was 8.75 × 10^−4^, and NW3 was 3.35 × 10^−4^.

Lee et al. [82] constructed a novel four-probe 3ω measurement platform. The thermal and electrical conductivities of a single nanowire were measured using the 3ω and 1ω signals that were produced using the four-probe approach. To quantify the Seebeck coefficient, a second nanoelectrode equipped with a nanoheater was also utilized. The bulk SiC’s *κ* value of 300 W/mK was higher than the single SiC nanowire’s value of 86.5 ± 3.5 W/mK. The *ZT* value of the single SiC nanowire was approximately 120 times higher than that of the bulk β-SiC. The 3ω method used in their study was based on the four-probe technique, which is a time-independent frequency domain technique that provides excellent signal-to-noise ratios using narrow-band detection techniques to eliminate spurious signals.

All the aforementioned approaches for optimizing thermoelectric properties are achieved by increasing the scattering of phonons at grain boundaries or interfaces to reduce thermal conductivity. In addition, numerous studies have been conducted to improve the Seebeck coefficients by constructing low-dimensional nanostructures, including nanosized [83,84] and quantum dot superlattices [85,86]. Low-dimensional SiC nanomaterials have superior mechanical properties, chemical stability, and thermoelectric properties compared to conventional bulk materials and have significant advantages and broad application prospects in thermoelectrics. More in-depth research is required on the use of low-dimensional SiC nanomaterials to obtain better thermoelectric effects.

Although SiC nanowires (SiC NWs) and thin films (SiC TFs) successfully diminish the thermal conductivity of SiC by efficiently scattering phonons at their interfaces, their fabrication processes are frequently cost-intensive and intricate. Nonetheless, the flexibility in tailoring the dimensions and orientations of SiC NWs presents avenues for decreasing thermal properties in targeted applications. Similarly, SiC superlattices, another promising candidate for reducing thermal conductivity, leverage their thin layer structure to effectively scatter phonons. However, akin to SiC NWs and TFs, their production and seamless integration pose formidable challenges. Moreover, the durability of these superlattices under extreme conditions necessitates rigorous research to guarantee consistent performance over prolonged durations.

Table 4 presents an interesting phenomenon: with an increase in porosity and a decrease in material dimensions, the thermal conductivity of the material shows a decreasing trend. However, in the same 1D SiC nanowires, the thermoelectric parameters are different, which may be due to differences in the measuring instruments and methods. Therefore, developing new low-dimensional measurement technologies for thermoelectric parameters is important because they can improve the accuracy and reliability of measurements and promote the wide application of thermoelectric materials in related fields.

### 3.3. Increase in Seebeck Coefficient

Typically, the Seebeck coefficient is described by the Pisarenko relationship [87,88]:

(12) $$S=\frac{8\pi^{2}K_{B}^{2}T}{3q\hbar^{2}}m_{d}^{*}{\left(\frac{\pi }{3p}\right)}^{2/3}$$

In the above equation, *K_B_* is the Boltzmann constant, *q* is the carrier charge, *p* is the hole concentration, *ħ* is the Planck constant, and $m_{d}^{*}$ is the DOS effective mass. The equation indicates that modulating the carrier concentration and DOS effective mass can change the Seebeck coefficient. Variations in the carrier concentration result in simultaneous changes in the Seebeck coefficient and conductivity. Therefore, improving $m_{d}^{*}$ is fundamental to improving *S* [89]. The increase in $m_{d}^{*}$ relies on energy-band engineering, which enables higher degeneracy in the original isolated energy bands while maintaining the shape of the unchanged bands. This causes the DOS to rise in the vicinity of the Fermi energy level, which significantly reduces $m_{d}^{*}$. Consequently, the Seebeck coefficient increases [90]. In addition, *S* can be enhanced by introducing energy barriers in the C_B_ or V_B_ via doping or constructing a heterojunction structure to introduce energy barriers on the surface of the material such that the carriers undergo energy filtering effects [91,92,93,94].

#### 3.3.1. Low Dimensionality

**Figure 8:**
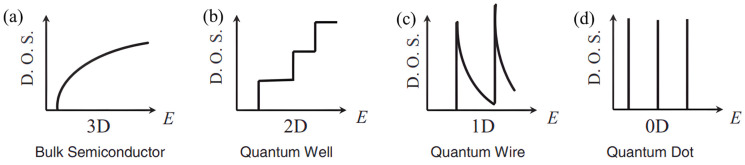
Electronic density of states for a (**a**) 3D bulk semiconductor, (**b**) 2D quantum well, (**c**) 1D nanowire, and (**d**) 0D quantum dot [93].

In 1993, Dresselhaus and Hicks [95] predicted that the DOS of thermoelectric materials would increase due to quantum confinement effects in 2D materials, which would raise the *PF*. Figure 8 illustrates how the quantum confinement effect causes a gradual discontinuity in the distribution of the Fermi energy levels of a material as its dimensionality decreases [93]. The more pronounced the discontinuous distribution of the Fermi levels, the higher the DOS, effective mass, and Seebeck coefficient of the thermoelectric material. As previously mentioned, low dimensionality boosts the interfacial phonon scattering of the material and can effectively reduce the thermal conductivity of the material while increasing the Seebeck coefficient, thereby significantly elevating the *ZT* value of the material [96]. This improvement in the thermoelectric properties of the material is achieved by eliminating the coupling between the thermoelectric parameters.

The commonly used methods for preparing 2D materials, chemical vapor deposition and physical vapor deposition, have difficulty meeting these requirements owing to their low deposition rates. Therefore, new techniques have been developed for the quick fabrication of SiC films, such as thermal plasma chemical vapor deposition, thermal plasma physical vapor deposition (TPPVD), and pyrolytic evaporation deposition. Wang et al. [97] used the TPPVD technique to prepare SiC films with a thickness of approximately 300 μm at a deposition rate of 300 nm/s or higher. The measurement range for the thermoelectric characteristics was 300–973 K using N_2_, B, and B_4_C as dopants. The absolute Seebeck coefficient value for the un-doped SiC thick film (480 μV/K) was higher than that for the sintered bulk SiC (361 μV/K). The specific thermoelectric parameters are listed in Table 5. A research team from Tsinghua University [98] successfully prepared low-doped Al nano-SiC films with grain sizes of 5–10 nm on silicon oxide substrates using the magnetron sputtering of SiC and Al targets. The highest obtained Seebeck coefficient was −393 μV/Km, and the lowest value of resistivity was 3.2 × 10^−4^ Ω·m. In the temperature range of 383–533 K, the absolute value of the *S*-ln*T* linear slope was 999 μV/K, which was larger than that of conventional semiconductors (129 μV/K).

#### 3.3.2. Heterojunction Structure

Nobel laureates Geim and Novoselov [99] proposed that by vertically stacking 2D monolayers into heterojunctions relying on van der Waals forces, the resulting heterojunction structures would yield new physical characteristics that are distinct from those of the respective 2D materials. Heterojunctions have been used in thermoelectrics for a relatively short period. The earliest available combination of heterostructures with thermoelectric materials was the P-Si/N-SnSe_2_ structure proposed by Hady in 1999 [100] that reduced the band gap to 0.96 eV after annealing and improved the thermoelectric efficiency of an otherwise single semiconductor. Recently, the interfacial design of heterojunctions to achieve the independent transport of electricity and heat has been increasingly recognized. Although electrical and thermal conductivities are difficult to separate in conventional designs, thin-film heterojunction materials with conductivity and thermal conductivity separated at the interface promote electrical conductivity. However, they suppress thermal transport in the heterojunction, enabling the independent regulation of electrical and thermal conductivities [101,102], with low thermal conductivity, high electrical conductivity, and the ability to concurrently improve the conductivity and Seebeck coefficient [103,104]. To date, few studies have reported using SiC and other semiconductor composites for constructing heterojunctions to improve the overall thermoelectric properties.

**Figure 9:**
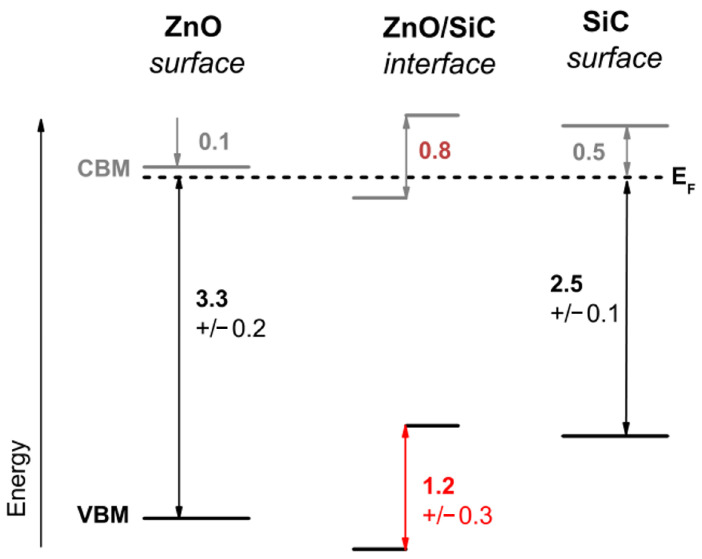
Energy-band diagram of ZnO/SiC. The middle number represents the band arrangement of the ZnO/SiC interfaces with interface-induced band bending [105]. All numbers are in eV.

In 2016, an extensive study of charge separation at the heterojunction interface was carried out by Zhang et al. [105]. They used molecular beam epitaxial deposition to cover n-type 6H-SiC single-crystal substrates with intrinsic n-type ZnO. At the ZnO/SiC interface, the V_B_ and C_B_ shifted by 1.2 ± 0.3 and 0.8 eV, respectively, which explained the excellent rectification properties of the homotypic ZnO/SiC heterojunction (Figure 9). Thus, a ZnO/SiC heterojunction was formed where bending of the energy band occurred. The electrons in the SiC C_B_ are more easily injected into the C_B_ of ZnO. Consequently, the holes from the V_B_ of ZnO migrate more easily to the V_B_ of SiC. ZnO/SiC heterojunctions increased carrier density and transport efficiency by facilitating the separation of interfacial charges. This discovery provided an important theoretical basis for an in-depth understanding of heterojunction structures.

**Figure 10:**
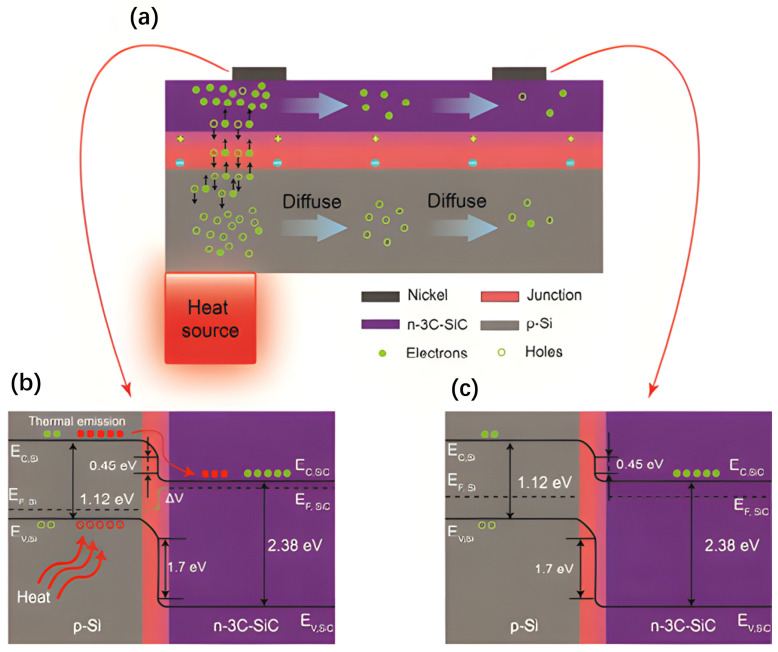
(**a**) Charge carrier concentration in the electrode, (**b**) energy-band diagram of the 3C-SiC/Si heterojunction *E_h_* electrode under heating conditions, and (**c**) energy-band diagram of the 3C-SiC/Si heterojunction *E_c_* under heating conditions [106].

In 2021, Guzman et al. [106] epitaxially grew n-type 3C-SiC (111) films doped with N atoms at a thickness of approximately 390 nm on a p-Si (100) substrate using a low-pressure chemical vapor deposition (LPCVD) reactor at 1000 °C. The surface of the 3C-SiC film was sputtered with a 300 nm thick layer of nickel. A 3C-SiC/Si heterojunction was formed by photolithographically patterning two Ni electrodes. At room temperature, the concentration of the thermally generated charge carriers was proportional to $\exp\left(-E_{a(d)}/K_{B}T\right)$, where $K_{B}$ is the Boltzmann constant, *T* is the thermodynamic temperature, and *E_a_*_(*d*)_ is the activation energy of the acceptor or donor. At higher temperatures, the concentration of charge carriers generated by the thermal energy was proportional to the $\exp\left(-E_{g}/2K_{B}T\right)$ of both materials, where *E_g_* is the bandgap. The increased energy in the form of heat produced more charge carriers in Si, and the electrons in the C_B_ of Si tended to transfer to the C_B_ of SiC, whereas the holes in the V_B_ of SiC tended to transfer to Si, as shown in Figure 10. This created electron–hole pairs in the heterostructure, which reduced the probability of their complexation and increased the charge migration efficiency, conductivity, and Seebeck coefficient. Figure 10b,c shows the heterojunction energy-band patterns of the hot and cold electrodes, respectively. They reported a Seebeck coefficient of −374.78 μV/K for the SiC/Si heterojunction, which was much higher than the Seebeck coefficients of polycrystalline (−108 μV/K) and single crystals (−30 μV/K).

Zeng et al. [107] improved the power factor of materials by changing layers and applying the stacking method of 2D-layered materials. This van der Waals stacked laminate material technique presents new avenues for the design of materials and property tuning. Cramer et al. [108,109] investigated the properties of n-type superlattice thin-film materials formed from alternating layers of Si and SiC. Thirty-one layers of 10-nm thick Si/SiC bilayer superlattices were deposited via high-speed ion-beam sputtering. To obtain the temperature-dependent thermoelectric properties of their sample, it was deposited on Si, quartz, and mullite substrates, and its thermoelectric properties were tested. The sample deposited on the Si substrate had similar resistance to the Si substrate, which rendered the measurements unreliable. For the Si/SiC multilayer systems on quartz and mullite substrates, the Seebeck coefficients were −2600 and −2300 μV/K at 870 K, while the *ZT* values were 0.08 and 0.07, respectively. Table 6 displays the resistivity and thermal conductivity of the films on quartz substrates were 3.4 × 10^−2^ Ω·m and 2 W/mK, respectively. This experiment further confirmed the theoretical research by Hicks et al. [95] in that the nanoscale superlattices showed certain quantum limiting effects and could increase the effective mass of carriers, Seebeck coefficient, and electrical conductivity while maintaining constant mobility. However, this phenomenon only occurs at high temperatures.

The thermoelectric performance data for the different SiC samples are summarized in Table 7. The absolute Seebeck coefficient value for these samples exceeded that of the sintered bulk SiC (361 μV/K) [97]. Notably, with the reduction in material dimensions and the building of heterojunction structures, the energy-band structure was optimized, which increased the Seebeck coefficient. This discovery provides a new path for the development of thermoelectric materials. As research continues, high-performance SiC heterojunction devices will emerge in the future, making significant contributions to the advancements in science and technology.

## 4. Conclusions and Outlook

Under high-temperature conditions and other extreme environments, thermoelectric conversion applications such as self-cooling devices, supersonic vehicles, and rockets have stringent requirements for temperature and for the properties of the thermoelectric materials [11,12]. Therefore, developing thermoelectric materials that can maintain excellent performance and high stability at high temperatures has become the focus of current research. As a wide-bandgap semiconductor, SiC has a lower probability of bipolar diffusion than traditional tellurium alloys and is more suitable for thermoelectric applications at high temperatures. Moreover, it has the advantages of low toxicity, good mechanical strength, high thermal and chemical stability, and abundant raw material elements, which facilitate its use as a high-temperature thermoelectric material. We have conducted a comprehensive and systematic review and in-depth comparison of existing and potential performance improvement strategies for SiC thermoelectric materials. This process covers various cutting-edge technology paths, from element or compound doping to innovative structural designs such as 3D porous SiC ceramics, 2D thin films, and 1D nanowires. In this paper, the specific contribution of each scheme to the thermoelectric properties of SiC and its intrinsic influence mechanism are discussed in detail, aiming to reveal the unique role of different strategies for optimizing material properties. Through rigorous quantitative analysis methods, we clearly define the advantages and potential limitations of each scheme to build a solid data framework and theoretical foundation for the parameter optimization of SiC thermoelectric materials. These achievements not only provide valuable guidance for future material design but also greatly promote the accuracy and efficiency of experimental verification, leading the research on SiC thermoelectric materials to higher performance and wider applications. Although these strategies have improved the thermoelectric performance of SiC, several challenges remain.

First, owing to the high proportion of covalent bonds in SiC, densifying it without using additives is difficult, and full densification can only be achieved under extreme conditions, such as ultra-high temperatures or pressures [110,111]. Therefore, reducing the sintering temperature of SiC thermoelectric materials is critical. Second, the preparation process for porous SiC materials is complex, and the shape and size of the material pores are difficult to control; these factors greatly affect the stability of the thermoelectric properties, diminishing practicability. Third, increasing thermoelectric performance by adding a second phase requires a uniform distribution of inclusions, and undesirable aggregation may occur if the second phase is not well dispersed in the main phase [112]. Further research is required to enhance the degree of particle dispersion. Fourth, low-dimensional SiC materials remain underexplored. The production of SiC NWs, TFs, and superlattices remains costly and complex, necessitating further research into cost-effective and scalable fabrication methods. Additionally, the long-term stability of these advanced structures in harsh environments must be thoroughly investigated to ensure reliable performance over extended periods. Future work should focus on overcoming these challenges, as well as exploring new materials and structures that can further enhance the thermoelectric properties of SiC, ultimately leading to the development of high-performance, cost-effective, and durable thermoelectric materials for use in extreme environments.

In this paper, we summarize strategies for improving the thermoelectric value of SiC. These include the use of energy-band engineering to design nanostructures, such as heterojunctions and superlattices, to optimize thermoelectric properties via quantum confinement effects. In future research, *ZT* can continue to be further improved via doping, nanostructural design, resonance energy levels, and energy-band engineering. Thus, thermoelectric energy conversion technology will be more widely used for the conversion of automobile and industrial waste heat.

## Figures and Tables

**Table 1:** Property comparisons of common SiC polytypes [27,28].

Property	6H-SiC	4H-SiC	3C-SiC
Crystal structure	Hexagonal	Hexagonal	Cubic
Band gap	3.0 eV	3.2 eV	2.3 eV
Lattice parameter	a = 0.3073 nm c = 1.5118 nm	a = 0.3073 nm c = 1.0053 nm	a = 0.4349 nm
Intrinsic carrier concentration at 300 K	10^−5^ cm^−3^	10^−7^ cm^−3^	10 cm^−3^
Saturated electron velocity	2.0 × 10^7^ cm/s	2.5 × 10^7^ cm/s	2.0 × 10^7^ cm/s
Dielectric constant	9.7	9.7	9.7
Thermal conductivity	300–500 W/mK	300–500 W/mK	300–500 W/mK

**Table 2:** Summary of the thermoelectric properties of SiC following doping and second-phase recombination.

	Dopants	Sintering Method	Doping Amount	*κ* (W/mK)	*n* (m^−3^)	*ρ* (Ω·m)	*S* (μV/K)	*PF* (W/mK^2^)	*Z* (K^−1^)
Comparison of Al-doped and N-doped SiC under the same conditions	Al_4_C_3_ [42,43]	SPS	3~10 wt%		7.6 × 10^24^			2 × 10^−5^	
	Si_3_N_4_ [41,42,43]				1.7 × 10^26^	3.3 × 10^−6^	−150	1.5 × 10^−4^	
Comparison of co-doping and single-element doping	AlN [53]	Atmospheric pressure sintering	0.5 wt%, 1 wt%, 3 wt%, 5 wt%	8.5		1 × 10^−4^	525		10^−5^
	Al_4_C_3_ [53]			8		10^−5.2^	400		10^−5^
N-type doping	Fe [44]	RF induction heating	1~20 wt%	16.5		1.8 × 10^−4^	−390		10^−5^
P-type doping	Ag [52]	Atmospheric pressure sintering	0.5~5 wt%	10^−3^	2.3 × 10^26^	5.6 × 10^−5^			4 × 10^−4^
Second-phase recombination	Ge [56]	RF induction heating	1~40 wt%	8		5 × 10^−4^	900		2 × 10^−5^

**Table 3:** Comparison of thermal conductivity between SiC film and bulk SiC.

Material	Thickness	Deposition Method	*κ* (W/mK)
Poly-SiC film [75] (N doped)	0.93 μm	LPCVD	64
SiC film [76]	0.498 μm	RF sputtering	1.44
SiC bulk [77]	10 cm	Hot-press sintering	270

**Table 4:** Summary of the characteristics of thermoelectric materials based on SiC in various dimensions.

Dimension	Dopant	Sintering Method	*κ* (W/mK)	*ZT*
3D	SiC bulk [69]	Hot-press sintering	120	3.65 × 10^−5^
	SiC foam [69]	Macromolecule pyrogenation combined with reaction bonding	30	1.338 × 10^−4^
2D	Poly-SiC film [75]	LPCVD	64	4.6 × 10^−6^
	SiC film [76]	RF sputtering	1.44	
1D	SiC nanowire [79]	Combustion in a calorimetric bomb	4–12	3.01 × 10^−3^–8.75 × 10^−4^
	SiC nanowire [82]	CVD	86.5 ± 3.5	0.12

**Table 5:** Thermoelectric parameters of the thick films of N_2_-, B-, and B_4_C-doped SiC prepared using the TPPVD method (973 K) [97].

Dopant	*ρ* (Ω·m)	*S* (μV/K)	*PF* (W/mK^2^)	*ZT*
Unadulterated	10^−2^ to 10^−3^	−480	1.6 × 10^−4^	0.01946
N_2_ doping	10^−4^ to 10^−5^		1 × 10^−3^	0.121625
B doping			1.65 × 10^−4^	0.020433
B_4_C doping		541	1.49 × 10^−4^	0.018487

**Table 6:** Thermoelectric parameters of Si/SiC multilayer systems on different substrates [108,109].

Substrate	*ρ* (Ω·m)	*n* (cm^−3^)	*S* (μV/K)	*κ* (W/mK)	*ZT*
Quartz	3.4 × 10^−2^	−3.2 × 10^17^	−2600	2	0.08
Mullite		−3.3 × 10^15^	−2300		0.07
Si	Electrical tests cannot be performed				

**Table 7:** Summary of the thermoelectric properties of SiC thin films.

Material	Sintering Method	*ρ* (Ω·m)	*S* (μV/K)	*ZT*
Thick SiC films [97]	TPPVD	10^−2^–10^−3^	−480	0.01946
Low-doped Al nano-SiC films [98]	Magnetron sputtering	3.2 × 10^−4^	−393	
SiC/Si heterojunction [106]	LPCVD		−374.78	
Si/SiC multilayer systems [108,109]	High-speed ion-beam sputtering deposition	3.4 × 10^−2^	−2600	0.08

## Data Availability

No new data were created or analyzed in this study. Data sharing is not applicable to this article.
